# Aligning Care With the Priorities of Rural Veterans: Evaluation of a PPC-Focused Geriatric Consultative Model

**DOI:** 10.1093/geroni/igaf122.2025

**Published:** 2025-12-31

**Authors:** Katherine Ritchey, Gwen Bernacki, Amy Thomas, Linnaea Schuttner, Karl Brown, Erica Martinez

**Affiliations:** VA Puget Sound Health Care System, Seattle, Washington, United States; VA Puget Sound Health Care System, Seattle, Washington, United States; VA Puget Sound Health Care System, Seattle, Washington, United States; VA Puget Sound Health Care System, Seattle, Washington, United States; VA Puget Sound Health Care System, Seattle, Washington, United States; Department of Veterans Affairs, Seattle, Washington, United States

## Abstract

Patient Priorities Care (PPC) is an effective approach to identifying and aligning ‘what matters’ with healthcare for patients with multiple chronic conditions (MCC). The enrollment of older, rurally-based veterans with MCC will increase throughout Puget Sound, placing additional strain on VA clinics lacking geriatric specialty care. Therefore, we developed a geriatric consultation that utilizes the PPC approach to improve the identification of rehabilitative or social services and support rural VA clinics in their ability to care for persons with MCC. Patients were referred to a Geriatric PPC Consultation clinic received recommendations to health care. Multiple linear regression was used to correlate total number of recommendations with Jen frailty (JFI) scores. Surveys and semi-structured interviews with providers, patients and/or caregivers explored experience with the consultation service. On average, patients received four recommendations per consult. Frailty scores had no effect on number of recommendations for both rural (p = 0.85) and non-rural (p = 0.35) groups. Veteran and care partner experience with the consultation was positive and enhanced the clinical-patient encounter. Results from surveys and interviews suggest that this approach may improve Veteran perception of person-centered care on aspects that ‘matter most’ and the quality of VA healthcare encounters. Most providers indicated that consultation increased veteran access to VA and community-based services and was work-load neutral. The PPC approach identifies unmet health care needs for rural, patients with MCC and is supportive to patients, care partners and providers. Future work is needed to clarify how to scale this model across VA.

